# Diverging trends of chronic bronchitis and smoking habits between 1998 and 2010

**DOI:** 10.1186/1465-9921-14-16

**Published:** 2013-02-08

**Authors:** Simone Accordini, Angelo Guido Corsico, Isa Cerveri, Leonardo Antonicelli, Francesco Attena, Roberto Bono, Lucio Casali, Marcello Ferrari, Alessandro Fois, Pierpaolo Marchetti, Pietro Pirina, Roberta Tassinari, Giuseppe Verlato, Roberto de Marco

**Affiliations:** 1 Unit of Epidemiology and Medical Statistics, Department of Public Health and Community Medicine, University of Verona, c/o Istituti Biologici II, Strada Le Grazie 8, 37134, Verona, Italy; 2 Division of Respiratory Diseases, IRCCS “San Matteo” Hospital Foundation, University of Pavia, Pavia, Italy; 3 Allergy Unit, Department of Internal Medicine, Immuno-Allergic and Respiratory Diseases, Ospedali Riuniti di Ancona, Ancona, Italy; 4 Department of Experimental Medicine, Seconda Università di Napoli, Napoli, Italy; 5 Department of Public Health and Microbiology, University of Turin, Turin, Italy; 6 Chair of Respiratory Diseases, University of Perugia, Perugia, Italy; 7 Section of Internal Medicine, University of Verona, Verona, Italy; 8 Institute of Respiratory Diseases, University of Sassari, Sassari, Italy

**Keywords:** Allergic rhinitis, Asthma, Chronic bronchitis, Cigarette smoking, Epidemiology

## Abstract

**Background:**

No study has been carried out on the time trend in the prevalence of chronic bronchitis (CB) in recent years, despite its clinical and epidemiological relevance. We evaluated the trend in CB prevalence during the past decade among young Italian adults.

**Methods:**

A screening questionnaire was mailed to general population samples of 20–44 year-old subjects in two cross-sectional surveys: the Italian Study on Asthma in Young Adults (ISAYA) (1998/2000; n = 18,873, 9 centres) and the screening stage of the Gene Environment Interactions in Respiratory Diseases (GEIRD) study (2007/2010; n = 10,494, 7 centres). CB was defined as having cough and phlegm on most days for a minimum of 3 months a year and for at least 2 successive years. The prevalence rates and the risk ratios (RRs) for the association between CB and each potential predictor were adjusted for gender, age, season of response, type of contact, cumulative response rate, and centre.

**Results:**

CB prevalence was 12.5% (95% CI: 12.1-12.9%) in 1998/2000 and 12.6% (95% CI: 11.7-13.7%) in 2007/2010; it increased among never smokers (from 7.6 to 9.1%, p = 0.003), current light smokers (<15 pack-years; from 15.1 to 18.6%, p < 0.001), and unemployed/retired subjects (from 14.3 to 19.1%, p = 0.001). In this decade, the prevalence of current smoking decreased (from 33.6 to 26.9%, p < 0.001), whereas the prevalence of unemployment/premature retirement (from 5.3 to 6.0%, p = 0.005), asthma (from 5.0 to 6.2%, p = 0.003), and allergic rhinitis (from 19.5 to 24.5%, p < 0.001) increased. In both 1998/2000 and 2007/2010, the likelihood of having CB was significantly higher for women, current smokers, asthmatic patients, and subjects with allergic rhinitis. During this period, the strength of the association between CB and current heavy smoking (≥15 pack-years) decreased (RR: from 4.82 to 3.57, p = 0.018), whereas it increased for unemployment/premature retirement (from 1.11 to 1.53, p = 0.019); no change was observed for gender, asthma, and allergic rhinitis.

**Conclusions:**

Despite the significant reduction in current smoking, CB prevalence did not vary among young Italian adults. The temporal pattern of CB prevalence can only be partly explained by the increase of unemployment/premature retirement, asthma and allergic rhinitis, and suggests that other factors could have played a role.

## Background

Chronic bronchitis (CB) (i.e. a mucus-producing cough on most days for a minimum of 3 months a year and for at least 2 consecutive years) is a highly prevalent respiratory disorder, usually associated with cigarette smoking [1,2]. CB can precede the development of chronic obstructive pulmonary disease (COPD) [3,4], and it increases the likelihood of exacerbations, hospitalization, and mortality in COPD patients [5-7]. Passive smoking, air pollution, and occupational exposures to dust, gas, and fumes can also increase the risk of CB [8-10]. Furthermore, CB may be associated with asthma and may be a marker of both asthma severity [11] and poor control of symptoms [12], which in turn are associated with a heavy socio-economic burden [13-15]. CB in asthmatic subjects may also indicate the presence of the asthma-COPD overlap syndrome, which is a difficult condition to treat [16]. In addition, CB can be related to allergic rhinitis and gastroesophageal reflux disease (GORD) [17,18].

While the prevalence of asthma and allergic rhinitis increased during the last decade in Italy [19], so far no study has been carried out on the time trend in the prevalence of CB in recent years, despite its clinical and epidemiological relevance. Accordingly, the present study is aimed at evaluating the ten-year trend in the prevalence of CB among young adults (20–44 years) in Italy, and at evaluating whether the pattern of the association with potential predictors changed during the last decade. To fulfil these objectives, we used the data from two cross-sectional surveys, the Italian Study on Asthma in Young Adults (ISAYA) [20] and the screening stage of the Gene Environment Interactions in Respiratory Diseases (GEIRD) study [21].

## Methods

### Design of the study and definitions

ISAYA and GEIRD-screening stage were two cross-sectional surveys on respiratory health in the general adult population, which were carried out in Italy between 1998–2000 and between 2007–2010, respectively. ISAYA involved 25,969 subjects from 9 centres (Ferrara, Pavia, Pisa, Sassari, Sassuolo, Siracusa, Turin, Udine, Verona) with an overall response rate of 72.7% [20], whereas GEIRD involved 18,357 subjects from 7 centres (Ancona, Pavia, Salerno, Sassari, Terni, Turin, Verona) with an overall response rate of 57.2% [19]. In both the studies, random samples of 3,000 subjects aged 20–44 (men/women ratio = 1) were selected in each centre from the general population, using the local health authority registers. A screening questionnaire [21,22] was mailed to each subject up to three times and then administered by telephone in case of non response. Ethics approval was obtained in each centre involved in GEIRD from the appropriate ethics committee (Comitato Etico dell’Azienda Ospedaliero-Universitaria Ospedali Riuniti di Ancona; Comitato di Bioetica della Fondazione IRCCS Policlinico San Matteo di Pavia; Comitato Etico dell’Azienda Sanitaria Locale SA/2 di Salerno; Comitato di Bioetica dell’Azienda Sanitaria Locale di Sassari; Comitato Etico delle Aziende Sanitarie dell'Umbria di Perugia; Comitato Etico dell’Azienda Sanitaria Locale TO/2 di Torino; Comitato Etico per la Sperimentazione dell'Azienda Ospedaliera Istituti Ospitalieri di Verona). All participants were fully informed about all aspects of the research project and consented to complete and return the questionnaire.

The screening questionnaires used in ISAYA and GEIRD shared a set of core questions (mainly taken from the European Community Respiratory Health Survey [ECRHS] questionnaires [23]) on the presence of asthma (self-report of the disease during lifetime with or without a physician diagnosis, frequency of asthma attacks and use of anti-asthmatic drugs in the last 12 months), asthma-like symptoms (wheezing, nocturnal tightness in the chest, and attacks of shortness of breath at night time in the last 12 months), allergic rhinitis (any nasal allergies, including hay fever), and CB (cough and phlegm on most days for a minimum of 3 months a year and for at least 2 successive years), as well as questions on smoking habits (the age of smoking commencement, the amount of tobacco smoked, and the age at which smokers stopped smoking). The screening questionnaire used in GEIRD also contained questions on the frequency of vehicular traffic (cars and trucks) near home.

Smoking habits were classified as current light/heavy smoking, past light/heavy smoking, or never smoking according to lifetime pack-years, considering 15 pack-years as the cut-off. Ever smoking was defined as having smoked at least one cigarette per day or one cigar a week for one year. Asthma was considered present if a subject had reported asthma confirmed by a doctor and at least one respiratory symptom (wheezing, nocturnal tightness in the chest, attacks of shortness of breath at night time) or at least one attack of asthma or use of medicines because of breathing problems in the last 12 months.

### Statistical analysis

The data from all the centres that participated in ISAYA and/or GEIRD were used in the analysis. Gender, age, season of response, type of contact, and cumulative response rate were considered as potential confounders because of differences in their distribution between the two surveys (Table 1). In particular, the cumulative response percentile rank was used: each subject was ordered by study and centre according to the date of response to the questionnaire, and then he/she was attributed the ratio between his/her rank and the number of eligible subjects [19].

**Table 1:** Distribution of the design variables

	**ISAYA** (**1998**/**2000**) **N** = **25**,**969**	**GEIRD** (**2007**/**2010**) **N** = **18**,**357**	**p**-value*
N° of responders (response rate, %)	18,873 (72.7)	10,494 (57.2)	<0.001
Season at time of the interview, %			<0.001
spring	18.5	41.0	
summer	16.4	22.1	
autumn	40.4	25.7	
winter	24.7	11.2	
Phone contact, %	27.9	11.8	<0.001
Females, %	50.7	52.3	0.008
Age (years), mean (sd)	33.1 (6.9)	34.7 (7.1)	<0.001

* p-value obtained by Pearson’s chi-squared test and two-sample t test with unequal variances.

Adjusted prevalence rates of CB were computed by logistic regression models. The models had an indicator of the presence of CB as the dependent variable and the study (GEIRD vs ISAYA), the potential confounders reported above, and centre as covariates. Centre was included in the models in order to partly control for the potential confounding effect of ecological-level variables, such as environmental factors. Since the subjects were nested into groups (the study -ISAYA or GEIRD- in which they participated, crossed by centre), clustered sandwich estimators of the variance were used. The prevalence rates in each survey were estimated by setting the distribution of gender, season of response, type of contact, and centre equal to the average distribution, and by setting age and the cumulative response rate equal to the overall mean [24], in order to make the ISAYA and GEIRD estimates comparable.

The association between gender, smoking habits, occupational status, asthma, allergic rhinitis, and the presence of CB was estimated by the risk ratios (RRs), separately for each survey. The RRs were computed by Poisson regression models with robust standard errors (obtained by the Huber/White/sandwich estimator of the variance) and no offset [25], and were adjusted for the potential confounding effect of the same variables as the prevalence rates. The RRs were also computed by including a dummy variable for the high frequency of vehicular traffic (i.e. a continuous passage of cars and/or trucks) near home in the GEIRD model.

The statistical analysis was performed using STATA software, release 12 (StataCorp, College Station, TX).

## Results

### Ten-year trend in the prevalence of CB

**Figure 1:**
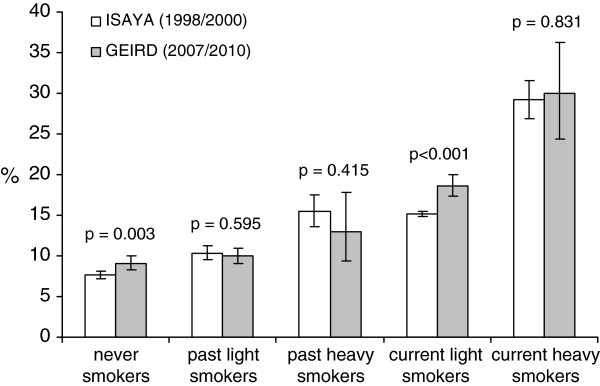
**Adjusted prevalence of CB according to smoking habits.** Bars represent 95% confidence intervals.

The prevalence of CB was 12.5% (95%CI: 12.1-12.9%) in 1998/2000 and 12.6% (95%CI: 11.7-13.7%) ten years later, and it did not significantly vary according to gender and age (Table 2). A statistically significant trend in the prevalence of CB was observed among never smokers (from 7.6 to 9.1%, p = 0.003) and current light smokers (<15 pack-years: from 15.1 to 18.6%, p < 0.001), but not among current heavy smokers (≥15 pack-years) and past smokers (Figure 1). According to occupational status, the prevalence of CB significantly increased among unemployed/retired subjects (from 14.3 to 19.1%, p = 0.001), but not among employed subjects/house-persons/students (from 12.3 to 12.1%, p = 0.764).

**Table 2:** Adjusted prevalence of CB according to gender and age

		**Adjusted prevalence(%)[95% CI]**		
		**ISAYA(1998**/**2000)**	**GEIRD(2007**/**2010)**	**p**-**value**^‡^
Gender*	Males	12.3 [12.0−12.6]	12.6 [11.8−13.4]	0.575
	Females	12.6 [11.9−13.3]	12.7 [11.3−14.3]	0.920
Age^†^	<30 years	11.8 [11.2−12.5]	12.2 [10.9−13.6]	0.577
	≥30 years	12.7 [12.2−13.2]	12.9 [12.0−13.8]	0.692
TOTAL^¶^		12.5 [12.1−12.9]	12.6 [11.7−13.7]	0.781

* prevalence obtained by stratifying on gender and adjusted for age, season of response, type of contact, cumulative response rate, and centre.

^†^ prevalence obtained by stratifying on age and adjusted for gender, season of response, type of contact, cumulative response rate, and centre.

^¶^ prevalence adjusted for gender, age, season of response, type of contact, cumulative response rate, and centre.

^‡^ p-value obtained by testing the null hypothesis that the regression coefficient of the dummy indicator of the study (GEIRD vs ISAYA) was zero.

### Ten-year trend in the distribution of smoking habits, occupational status, asthma, and allergic rhinitis

During this period, there was a statistically significant decrease in the percentage of current smokers (from 33.6 to 26.9%, p < 0.001) and in the percentage of current smokers according to the amount of exposure (current light smokers: from 22.5 to 19.8%, p < 0.001; current heavy smokers: from 7.0 to 4.2%, p < 0.001) (Table 3). Moreover, a statistically significant change was observed in the percentage of past heavy smokers (from 1.2 to 0.8%, p < 0.001), but not in the percentage of past light smokers. In addition, a statistically significant increase was observed in the percentage of unemployed/retired individuals (from 5.3 to 6.0%, p = 0.005), asthmatic patients (from 5.0 to 6.2%, p = 0.003), and subjects with allergic rhinitis (from 19.5 to 24.5%, p < 0.001). In the same decade, there was no statistically significant change in the percentage of asthmatic patients who also reported CB, and in the percentage of subjects with allergic rhinitis and coexisting CB (Table 4).

**Table 3:** Adjusted percentage* **of past**/**current smokers**, **unemployed**/**retired subjects**, **subjects with asthma**, **and subjects with allergic rhinitis**

	**Adjusted%[95% CI]**		
	**ISAYA(1998/2000)**	**GEIRD(2007/2010)**	**p**-**value**^†^
Past light smokers	11.2 [10.9−11.6]	11.7 [11.1−12.2]	0.219
Past heavy smokers	1.2 [1.1−1.3]	0.8 [0.7−1.0]	<0.001
Current light smokers	22.5 [22.0−23.1]	19.8 [18.9−20.7]	<0.001
Current heavy smokers	7.0 [6.7−7.4]	4.2 [3.8−4.6]	<0.001
Unemployed/retired subjects	5.3 [5.1−5.6]	6.0 [5.7−6.3]	0.005
Subjects with asthma	5.0 [4.7−5.3]	6.2 [5.6−6.8]	0.003
Subjects with allergic rhinitis	19.5 [19.2−19.9]	24.5 [23.8−25.2]	<0.001

* adjusted for gender, age, season of response, type of contact, cumulative response rate, and centre.

^†^ p-value obtained by testing the null hypothesis that the regression coefficient of the dummy indicator of the study (GEIRD vs ISAYA) was zero.

**Table 4:** Adjusted prevalence of CB according to the presence of asthma and allergic rhinitis

		**Adjusted prevalence(%)[95% CI]**		
		**ISAYA(1998/2000)**	**GEIRD(2007/2010)**	**p**-**value**^†^
Asthma*	Absent	11.0 [10.5−11.5]	10.7 [9.7−11.8]	0.665
	Present	31.2 [29.9−32.5]	33.3 [31.5−35.1]	0.144
Allergic rhinitis*	Absent	10.5 [10.2−10.9]	9.7 [8.8−10.6]	0.124
	Present	19.9 [19.0−20.8]	21.2 [20.0−22.5]	0.163

* prevalence obtained by stratifying on asthma/allergic rhinitis and adjusted for gender, age, season of response, type of contact, cumulative response rate, and centre.

^†^ p-value obtained by testing the null hypothesis that the regression coefficient of the dummy indicator of the study (GEIRD vs ISAYA) was zero.

### Changes in the pattern of the association between the presence of CB and the potential predictors

In both 1998/2000 and 2007/2010, the likelihood of having CB was significantly higher for women, current smokers and past heavy smokers (as compared to never smokers), asthmatic patients, and subjects with allergic rhinitis (Table 5). During this ten-year period, the strength of the association between smoking habits and the presence of CB decreased (with a dissimilar change in the RRs according to pack-years for both past and current smoking), whereas the strength of the association increased for unemployment/premature retirement. No change in the RRs was observed for gender, asthma, and allergic rhinitis. When the high frequency of vehicular traffic near home was also considered in the GEIRD model (2007/2010), traffic-related air pollution was significantly associated with the presence of CB (RR = 1.27; 95%CI: 1.14-1.41).

**Table 5:** **Mutually adjusted risk ratios** (**RRs**)* **for the association between CB and each potential predictor**

	**RR[95% CI]**		
	**ISAYA (1998/2000)**	**GEIRD (2007/2010)**	**p**-**value for heterogeneity**^†^
Gender (female vs male)	1.17 [1.09−1.27]	1.11 [1.00−1.22]	0.721
Smoking habits (vs never smoking):			
Past light smoking	1.40 [1.21−1.62]	1.03 [0.86−1.23]	0.015
Past heavy smoking	2.47 [1.96−3.13]	1.66 [1.19−2.31]	0.151
Current light smoking	2.15 [1.95−2.37]	1.95 [1.73−2.19]	0.144
Current heavy smoking	4.82 [4.31−5.39]	3.57 [3.11−4.11]	0.018
Occupational status (unemployed/retired subject vs employed subject/house-person/student)	1.11 [0.97−1.28]	1.53 [1.31−1.79]	0.019
Asthma (present vs absent)	2.08 [1.84−2.34]	2.01 [1.77−2.30]	0.635
Allergic rhinitis (present vs absent)	1.76 [1.60−1.93]	1.81 [1.63−2.02]	0.680

* RRs also adjusted for age, season of response, type of contact, cumulative response rate, and centre.

^†^ p-value obtained by considering the data from both the studies in the model and by testing the null hypothesis that the regression coefficient of the interaction term between the covariate and the dummy indicator of the study (GEIRD vs ISAYA) was zero.

### Sensitivity analysis

These results were confirmed when only the data from the four centres (Pavia, Sassari, Turin, Verona) that had participated in both the surveys were considered in the analysis. In particular, the prevalence of CB was 12.4% in 1998/2000 and 12.5% in 2007/2010, and the estimates of the RRs were comparable to those obtained in the main analysis (an additional table shows this in more detail [see Additional file 1]).

## Discussion

The main results of the present study are the following:

· the prevalence of CB did not vary during the past decade among young adults in Italy, despite a 7% reduction in the percentage of current smokers;

· the increase in the prevalence of CB among never smokers and the decrease in the strength of the association between current smoking and CB observed in the last ten years, suggest that factors other than smoking could have played a major role in determining the trend in the prevalence of CB;

· CB is common in asthmatic patients and in subjects with allergic rhinitis, and its association with these respiratory diseases has not changed with time.

### The prevalence of CB did not change in the past decade, despite the striking reduction in the percentage of current smokers

The prevalence rates of CB among 20–44 year-old subjects remained stable during the past ten years in Italy and were similar to that reported in the three Italian centres (Pavia, Turin, Verona) that had participated in the ECRHS in 1991/1993 (median prevalence: 11.3%) [17]. Moreover, the estimates from ISAYA and GEIRD are comparable to that obtained for the same age class in 16 industrialized countries in the ECRHS (10.2%) [17]. The impressive prevalence rates of CB estimated from the ISAYA and GEIRD studies enlighten the fact that CB is a substantial health problem even in young adults. Although CB may not lead to COPD in many of these subjects, our estimates suggest that the base of the COPD iceberg was wide and did not decrease during the past decade in Italy [2].

It could be surprising that there was no significant decrease in the prevalence of CB during the past decades in view of the fact that the percentage of current smokers decreased in Italy during the last twenty years [26], as also documented in our study. In fact, the relationship between smoking and CB is well known, and the amount of tobacco smoke determines the frequency and severity of symptoms [2,27]. Accordingly, we found that smoking habits is the strongest predictor of the presence of CB, since the RRs were the highest for current smoking. In addition, the strength of the association increased according to the amount of past and current active exposure. However, the prevalence of CB significantly increased among never smokers during the past decade, resulting in a reduction of the strength of the association between CB and smoking. Hence, our findings add to the emerging evidence that factors other than smoking play a major role in respiratory diseases [28].

### Temporal variation in the association between the presence of CB and other factors

The risk of having CB among unemployed/retired subjects significantly increased during the past decade. Unemployment/premature retirement can be considered a proxy measure for socio-economic status. Socio-economically disadvantaged subjects might be more susceptible to respiratory infections, such as pertussis, repeated viral infections, and tuberculosis. Moreover, the observed association could be due to factors, such as residential and workplace pollutant exposures [29], which affect respiratory health and which are more frequent in lower social classes, or could reflect a cumulative life course disadvantage [30]. The high prevalence of CB among unemployed/retired subjects in 2007/2010 supports the need for public health programs focused on vulnerable populations. In fact, the relationship between good health and sustaining employment strengthened during the last decades due to decreasing employment rates and increasing economic inactivity rates among subjects with poor health [31].

The strength of the association between the presence of CB and gender has not significantly changed during the past decade in Italy, and females showed a higher risk of having CB as compared to males, regardless of their smoking habits and socio-economic status, and the coexistence of asthma and allergic rhinitis. A possible explanation is that females may have more sensitive cough receptors than males [32]. Moreover, sex hormones may have an effect on airway reactivity [33].

The time trend in the prevalence of CB could have been influenced by factors other than smoking habits or socio-economic status, which were not measured in the present study. In fact, many causes may account for CB, especially in never smokers. In particular, we were not able to investigate the association with: i) passive smoking exposure, which is still common and it has been found to be significantly related to all types of respiratory symptoms [34]; ii) occupational exposures to dust, gas, and fumes over a long period of time, which has been documented to be a cause of CB [10]; iii) GORD [17,18], which is due to the fact that the acid reflux consumes the airways or the bronchi and triggers the body to create increased levels of mucus.

Finally, the exposure to outdoor air pollution was only investigated in 2007/2010 using a proxy variable of traffic-related air pollution near home. However, the observed association between vehicular traffic and the presence of CB supports the evidence that long-term exposure to air pollution is causally associated with respiratory illness [35,36]. In particular, living close to traffic has been documented to be associated with the prevalence of CB, which indicates that traffic-related air pollution has long-term effects on chronic respiratory disease [9,37]. Thus, in addition to the relatively rare episodes of incidental, heavy air pollution, the common levels of exposure to air pollutants may increase the prevalence of CB and other respiratory diseases.

### CB, asthma, and allergic rhinitis

The prevalence of asthma and allergic rhinitis increased in the last ten years in Italy, as already reported in a previous analysis of the ISAYA and GEIRD data [19]. Proposed contributing factors to the increase in the prevalence of asthma are the exposure to air pollution, infections, microbial substances in the environment, and obesity [38], whereas the upward trend in the prevalence of allergic rhinitis could be due to increasing air pollution, indoor environmental factors, improved hygiene practices, geo-climatic factors, or all of the above [19].

Despite the positive trend in their prevalence, the strength of the association with the presence of CB did not change for both asthma and allergic rhinitis, and our estimates are comparable to the results obtained in 16 industrialized countries in the ECRHS [17]. As already reported, the association between asthma and CB identifies a subgroup of patients characterized by frequent exacerbations, inadequate treatment, or poor disease control [11,12], which result in a heavy socio-economic burden [13-15]. Alternatively, it may be a result of the coexistence of asthma with COPD [16].

In agreement with previous studies [17,39], CB was highly prevalent among subjects with allergic rhinitis. This association confirms that post-nasal drip is a frequent cause of cough, which could also contribute to the volume of sputum expectoration. In particular, allergic rhinitis is a risk factor for sinusitis [40], which is highly prevalent in the general Italian population [41] and is a cause of post-nasal drip. However, especially in this case, CB may be multifactorial because many patients with asthma have rhinitis and, in turn, many patients with rhinitis have asthma or are at risk of asthma [42,43].

### Strengths and weaknesses of the study

The temporal change in CB is likely to reflect the trend in the prevalence rather than differences in the study design or changes in health care practice. In fact, our results were obtained by analyzing the data from two large surveys, which were carried out in the general population by using the same design, sampling frame, and protocol. Moreover, our estimates are based on self-reported symptoms, which were collected throughout international validated questionnaires [23] and are less influenced by diagnostic procedures.

A few caveats should be taken into account when interpreting our results. The Italian centres participating in ISAYA or GEIRD were not chosen randomly, but on the availability of research teams that were able to carry out the survey, and only a sub-sample of the Italian centres (Pavia, Sassari, Turin, Verona) was involved in both the surveys. However, despite this non-random selection, the study centres are located all over Italy and are representative of the geographical and climatic features of the country. Moreover, the sensitivity analysis carried out by considering only these four centres gave the same results as that obtained in the main analysis.

The participation rate declined from 72.7% in 1998/2000 to 57.2% in 2007/2010, as observed in other epidemiological studies over the past decades [44], and this decrease may have biased our estimates. In fact, symptom prevalence may be overestimated in GEIRD if the willingness to respond to the questionnaire is related to respiratory health [45]. However, it has been suggested that declines in participation rates are not likely to have a substantial influence on the exposure-disease associations or on the point estimates of measures of interest [44].

## Conclusions

Despite the significant reduction in current smoking, the prevalence of CB did not vary during the past decade among young adults in Italy. The temporal pattern in the prevalence of CB can only be partly explained by the increase in the prevalence of unemployment/premature retirement, asthma, and allergic rhinitis in the same period, and suggests that other factors could have played a role.

## Abbreviations

CB: Chronic bronchitis; COPD: Chronic obstructive pulmonary disease; GORD: Gastroesophageal reflux disease; ISAYA: Italian study on asthma in young adults; GEIRD: Gene environment interactions in respiratory diseases; ECRHS: European community respiratory health survey; RR: Risk ratio; 95%CI: 95% confidence interval.

## Competing interests

RdM, AGC, LC, MF, and PP have received a grant for the clinical stage of the GEIRD study from Chiesi Pharmaceuticals. LC has received a reimbursement for attending a symposium from Sigma-Tau Pharmaceuticals and a fee for organising education from Chiesi Pharmaceuticals. All remaining authors declare that they have no competing interests.

## Authors’ contributions

SA and RdM designed the study. SA, RdM, and AGC prepared the draft of the manuscript. SA did the statistical analysis. All the authors collected the data in their own centre and contributed to the interpretation of results and to the revision of the manuscript. All the authors read and approved the final manuscript.

## Supplementary Material

Additional file 1: Table E1 Mutually adjusted RRs for the association between CB and each potential predictor. The RRs were obtained by considering only the data from the four centres (Pavia, Sassari, Turin, Verona) that had participated in both the surveys. Click here for file
