# Supplementary material for: Diverging trends of chronic bronchitis and smoking habits between 1998 and 2010
Source: Respir Res. 2013 Feb 8;14(1):16. doi: 10.1186/1465-9921-14-16 (PMC3574861; doi:10.1186/1465-9921-14-16)
Supplement: Additional file 1: Table E1 — Mutually adjusted RRs for the association between CB and each potential predictor. The RRs were obtained by considering only the data from the four centres (Pavia, Sassari, Turin, Verona) that had participated in both the surveys. [file 1465-9921-14-16-S1.pdf]

ADDITIONAL FILE 1

Title: Diverging trends of chronic bronchitis and smoking habits between 1998 and 2010.

Authors: [1]Simone Accordini, [2]Angelo Guido Corsico, [2]Isa Cerveri, [3]Leonardo Antonicelli, [4]Francesco Attena, [5]Roberto Bono, [6]Lucio Casali, [7]Marcello Ferrari, [8]Alessandro Fois, [1]Pierpaolo Marchetti, [8]Pietro Pirina, [5]Roberta Tassinari, [1]Giuseppe Verlato, [1]Roberto de Marco.

Institutional affiliation of each author: [1]Unit of Epidemiology and Medical Statistics, Department of Public Health and Community Medicine, University of Verona, Verona, Italy; [2]Division of Respiratory Diseases, IRCCS "San Matteo" Hospital Foundation, University of Pavia, Pavia, Italy; [3]Allergy Unit, Department of Internal Medicine, Immuno-Allergic and Respiratory Diseases, Ospedali Riuniti di Ancona, Ancona, Italy; [4]Department of Experimental Medicine, Seconda Università di Napoli, Napoli, Italy; [5]Department of Public Health and Microbiology, University of Turin, Turin, Italy; [6]Chair of Respiratory Diseases, University of Perugia, Perugia, Italy; [7]Section of Internal Medicine, University of Verona, Verona, Italy; [8]Institute of Respiratory Diseases, University of Sassari, Sassari, Italy.

Table E1. Mutually adjusted risk ratios (RRs)* for the association between CB and each potential predictor. Only the data from the four centres (Pavia, Sassari, Turin, Verona) that had participated in both the surveys were considered.

	RR [95%CI]		
	ISAYA (1998/2000)	GEIRD (2007/2010)	p-value for heterogeneity†
Gender (female vs male)	1.21 [1.08–1.36]	1.11 [0.97–1.28]	0.816
Smoking habits (vs never smoking):			
Past light smoking	1.48 [1.20–1.83]	1.07 [0.85–1.36]	0.078
Past heavy smoking	2.82 [2.02–3.94]	1.43 [0.86–2.38]	0.076
Current light smoking	2.25 [1.95–2.60]	2.04 [1.72–2.41]	0.298
Current heavy smoking	5.52 [4.69–6.49]	3.46 [2.83–4.24]	0.005
Occupational status (unemployed/retired subject vs employed subject/house-person/student)	1.08 [0.89–1.31]	1.52 [1.23–1.89]	0.105
Asthma (present vs absent)	2.14 [1.80–2.55]	2.04 [1.69–2.45]	0.842
Allergic rhinitis (present vs absent)	1.71 [1.50–1.95]	1.92 [1.65–2.24]	0.223

* RRs also adjusted for age, season of response, type of contact, cumulative response rate, and centre

† p-value obtained by considering the data from both the studies in the model and by testing the null hypothesis that the regression coefficient of the interaction term between the covariate and the dummy indicator of the study (GEIRD vs ISAYA) was zero
